# Supplementary material for: Osmanthus-Loaded PVP/PVA Hydrogel Inhibits the Proliferation and Migration of Oral Squamous Cell Carcinoma Cells CAL-27
Source: Polymers (Basel). 2022 Dec 9;14(24):5399. doi: 10.3390/polym14245399 (PMC9784822; doi:10.3390/polym14245399)
Supplement: Supplementary file 1 [file polymers-14-05399-s001.zip › polymers-2057096-supplementary.pdf]

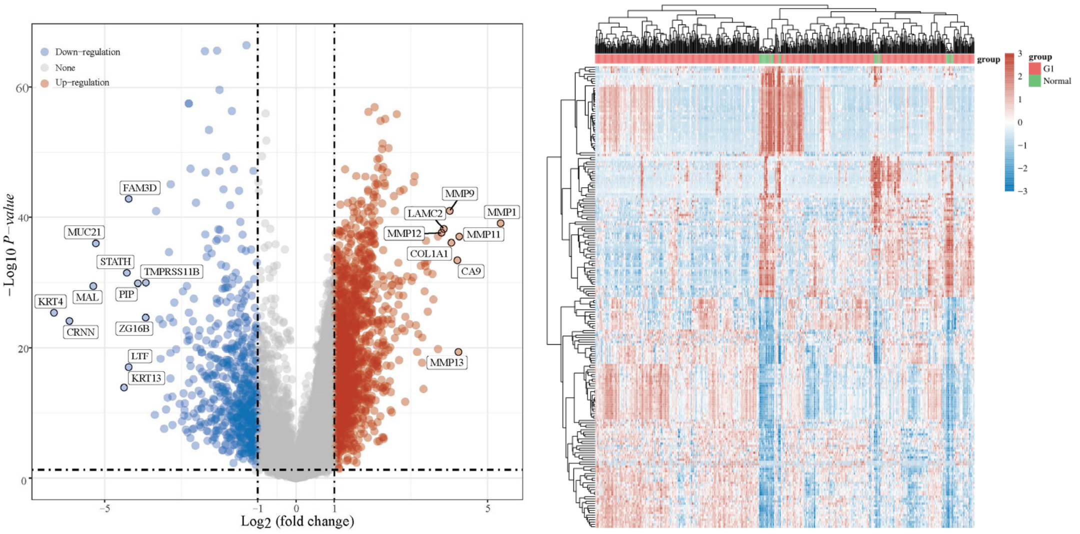

(A)

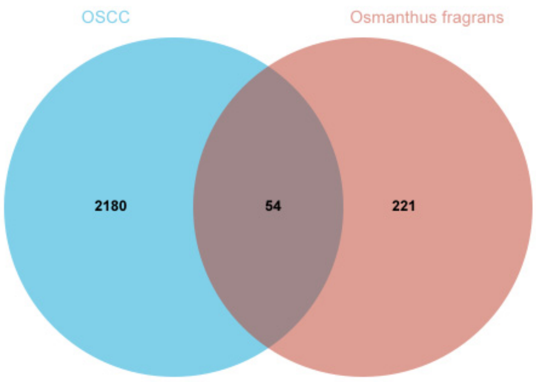

(B)

Figure S1. A. The differential genes of OSCC. Blue: down-regulated genes, Red: up-regulated genes, gray: undifferentially expressed genes. B. Identification of overlapping genes of differential genes of OSCC and osmanthus targeted genes.

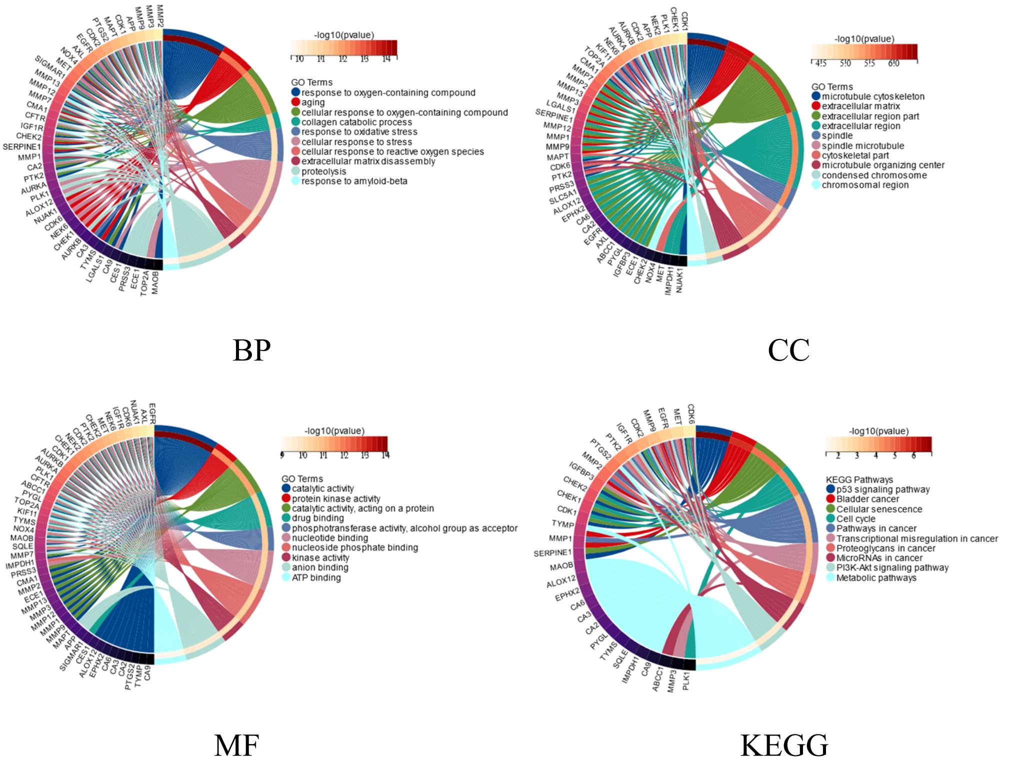

Figure S2. GO enrichment and KEGG pathway analysis represented in a chord plot. A. biological processes; B. cellular components; C. molecular functions; D. signaling pathways.

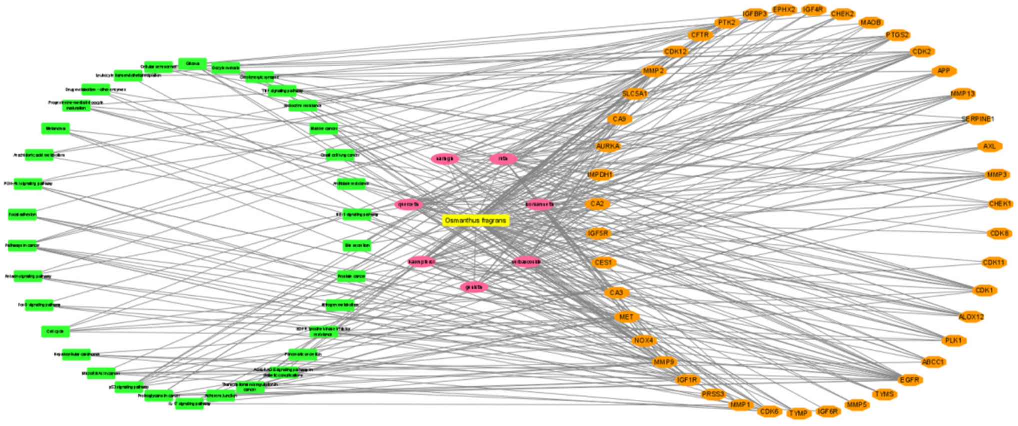

Figure S3. Component-Target-Pathway. Yellow: Osmanthus fragrans extract; Red: 7 main components; Orange: 54 overlapping genes; Green: Related KEGG pathway.

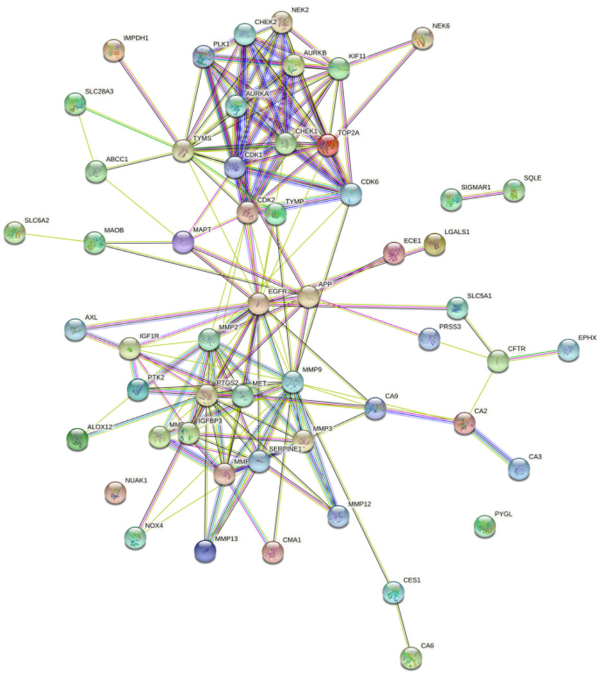

Figure S4. The PPI network analyzed by visualized by Cytoscape software. PPI network of 54 overlapping genes between differential genes of OSCC and osmanthus targeted genes.

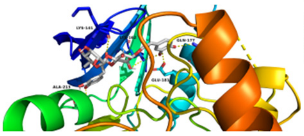

Acteoside

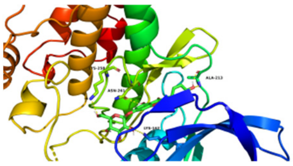

Genistein

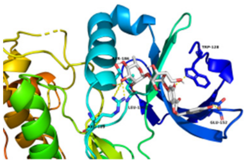

Hesperidin

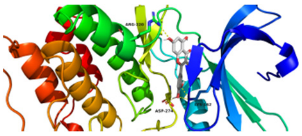

Isorhamnetin

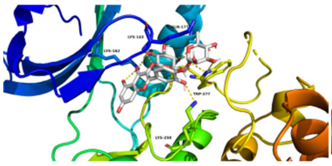

Rutinum

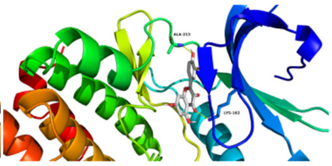

Quercetin

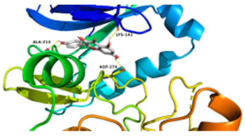

Kaempferol

(A)

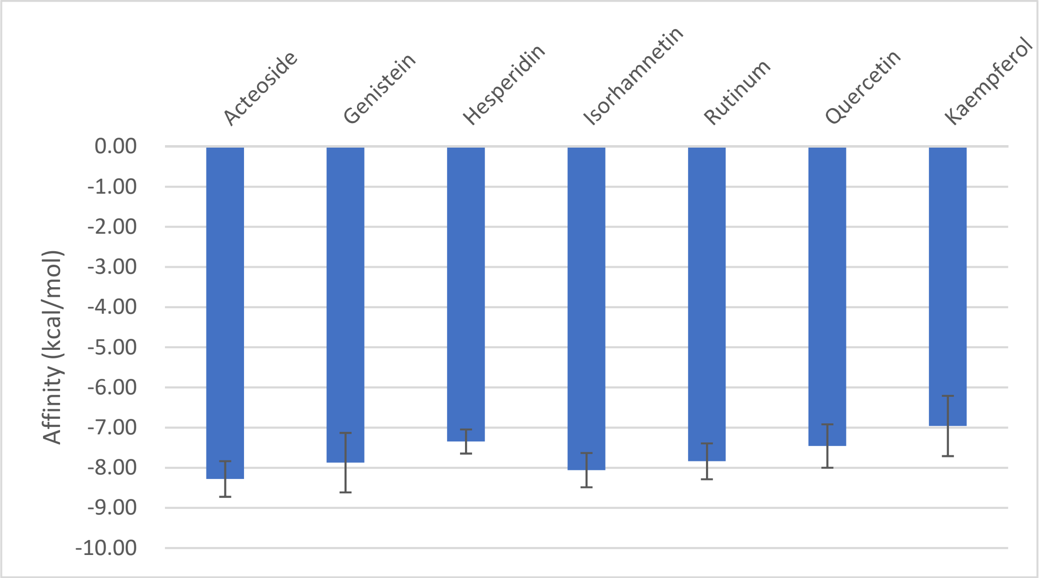

(B)

Figure S5. Molecular docking models of osmanthus extract and AURKA. A. 7 main components of osmanthus extract were docked with AURKA; B. the affinity of each component with AURKA.
